# Supplementary material for: Adolescent readiness for mobile mental health support in Soweto: A mixed-methods study
Source: Glob Ment Health (Camb). 2026 Feb 18;13:e40. doi: 10.1017/gmh.2026.10154 (PMC12964066; doi:10.1017/gmh.2026.10154)
Supplement: Madonsela et al. supplementary material [file S205442512610154Xsup001.docx]

Qualitative interview guide for focus group discussions

**Mental health understanding and health-seeking behaviour**

1. What do young people in your community say about mental health issues?
2. What is your experience of using mental health services from your local clinic or hospital?
3. Do you/why do you think it is important for adolescents in your community to have mental health care services tailored for them?

**Mobile-mental health use**

1. What are your thoughts about using mobile phones to access mental health services/seek help for your mental health needs?
2. How easy do you think it will be for you and your peers to use your mobile phones for mental health needs?
3. How difficult do you think it will be for you and your peers to use your mobile phones for mental health needs? (Internet connection, data affordability, compatible smartphone etc.)
4. Do you share your mobile phone with a parent/guardian? (Do you think this will affect how you engage with MMH?
5. Who can access your phone (do you find yourself hiding certain things on your phone as a result)?
6. What do you think will make it easy for adolescents in your community to use their mobile phones for their mental health needs?
7. What might be the benefits of using mobile phones for your mental health needs?
